# Magnetic Nanoemulsions: Comparison between Nanoemulsions Formed by Ultrasonication and by Spontaneous Emulsification

**DOI:** 10.3390/nano7070190

**Published:** 2017-07-22

**Authors:** Nathalia Rodríguez-Burneo, Maria Antònia Busquets, Joan Estelrich

**Affiliations:** 1 Department of Pharmacy, Pharmaceutical Technology and Physical Chemistry, Faculty of Pharmacy and Food Sciences, University of Barcelona, Avda. Joan XXIII, 27–31, 08028 Barcelona, Spain; nrodribu9@alumnes.ub.edu (N.R.-B.); mabusquetsvinas@ub.edu (M.A.B.); 2 Nanoscience and Nanotechnology Institute (IN2UB), Avda. Joan XXIII, 27–31, 08028 Barcelona, Spain

**Keywords:** nanoemulsion, oil olive, magnetic particles, indomethacin, spontaneous emulsification, low-energy method, high-energy method

## Abstract

Nanoemulsions are particularly suitable as a platform in the development of delivery systems. The type of nanoemulsion with a higher stability will offer an advantage in the preparation of a delivery system for lipophilic drugs. Nanoemulsions can be fabricated by different processing methods, which are usually categorized as either high- or low-energy methods. In this study, a comparison between two methods of preparing magnetic oil-in-water (O/W) nanoemulsions is described. The nanoemulsions were formed by sonication (the high-energy method) or by spontaneous emulsification (the low-energy method). In both cases, the oil phase was olive oil, and a phospholipid and a pegylated phospholipid were used as emulsifiers. To favor the comparison, the amounts of the components were the same in both kinds of nanoemulsions. Moreover, nanoemulsions were loaded with hydrophobic superparamagnetic nanoparticles and indomethacin. In vitro, releases studies indicated a short drug burst period followed by a prolonged phase of dissolutive drug release. The Korsmeyer-Peppas model can fit the associated kinetics. The results showed that such nanoemulsions are suitable as a platform in the development of delivering systems for lipophilic drugs. The long-term stability was also examined at different temperatures, as well as the interaction with plasma proteins. Nanoemulsion obtained by the low-energy method showed a great stability at 4 °C and at ambient temperature. Its size and polydispersity did not change over more than two months. The spontaneous emulsification method therefore has great potential for forming nanoemulsion-based delivery systems.

## 1. Introduction

Nanoemulsions can be recognized and defined as emulsions consisting of nanoscaled oil or water droplets (typically in the range 20–200 nm) dispersed in the external phase of opposite polarity by the effect of surfactant arranging at the oil/water interface [1,2]. In this way, two main types of nanoemulsions can be obtained: oil-in-water (O/W) and water-in-oil (W/O) nanoemulsions. Although both types present a large number of applications in different industrial fields, because of their exceptional properties (such as large surface area and robust stability), O/W nanoemulsions have applications in the food, personal care, and pharmaceutical industries. These nanoemulsions are commonly used in drug delivery research to administer hydrophobic drugs [3,4,5,6,7], development of healthy food drinks with hydrophobic nutrients [8,9,10,11,12], and preparation of skincare products [13]. In addition, nanoemulsion systems have potential for green chemistry, analogous to microemulsions as reaction media for decontaminating materials containing toxic organic compounds [14]. Hydrophobic magnetic nanoparticles can be incorporated into the oil droplets of O/W nanoemulsions generating a magnetic nanoemulsion. When the size of the magnetic nanoparticles is less than a critical value (the critical diameter), the magnetic structure of the material is a single domain. Magnetic nanoparticles formed by single domains are suitable for generating heat when an alternating magnetic field is applied. Further reduction in size, below the superparamagnetic diameter, makes the iron material superparamagnetic [15]. In a superparamagnetic material, the material is magnetized (the magnetic property—the magnetic moment by unit volume—is defined as magnetization) when an external magnetic field is applied, but revert to a non-magnetic state when the external magnet is removed. This is of great importance when the magnetic nanoparticles must be introduced into living systems (e.g., in drug delivery or magnetic hyperthermia), because, once the external magnetic field is removed, the magnetization disappears. In magnetic nanoemulsions, one must add the main property of the magnetic nanoparticles to the intrinsic properties of such colloidal systems: they can be guided to a desired zone in the organism with the aid of a magnetic field. The preparation of magnetic colloids began with the well-known synthesis of ferrofluid based on ferric and ferrous salts precipitation using alkaline medium [16]. Since that time, several types of magnetic colloids have been prepared: microspheres, latexes, and liposomes [17,18]. However, the properties of magnetic nanoemulsions have not been exploited for numerous applications; to the date, only the preparation of magnetic nanoemulsions as a tool for nucleic acid extraction [19], for detecting cations [20], and for non-enzymatic glucose detection [21] have been described.

Nanoemulsions are thermodynamically unstable systems; for this reason, energy input is required for their formation. Two main approaches are currently used for the preparation of nanoemulsions: high energy methods and low energy methods. For high energy methods, intense mechanical energy input is carried out by extreme shear stirring, high-pressure homogenizers, or ultrasounds [22,23,24]. For low energy methods, the chemical energy stored in the components is used by changing the spontaneous curvature of the surfactants. The modification of the curvature can be achieved by changing the temperature at constant composition, or changing the emulsion composition and/or environment conditions (e.g., temperature, pH, and ionic strength) [25]. The low energy methods include phase inversion methods, such as phase inversion temperature (PIT), phase inversion composition (PIC), emulsion inversion point (EIP), and spontaneous emulsification. Both approaches—high and low energy methods—present advantages and limitations, including requirements of only certain types of emulsifiers and oils and a large amount of surfactant, use of organic solvents, and low capacity of oil solubilization [26].

**Figure 1:**
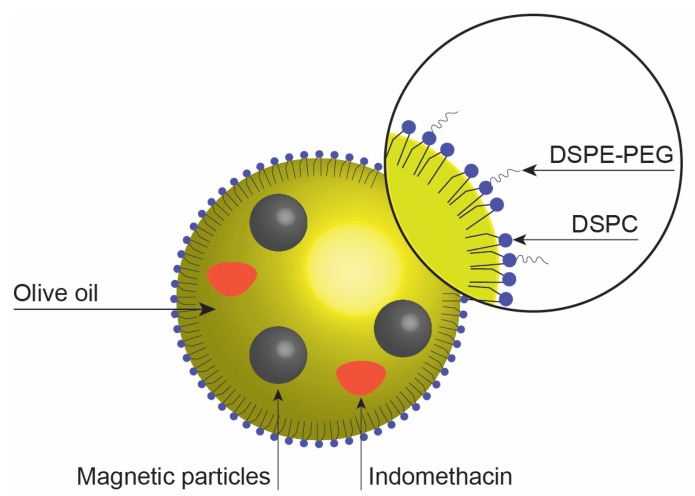
Schematic representation of a droplet of any magnetic nanoemulsion.

In the current study, we explore the influence of the method of preparation on the physicochemical properties and stability of magnetic nanoemulsions. The nanoemulsion is an oil-in-water emulsion formed from olive oil, oleic acid coated iron oxide nanoparticles, two phospholipids (1,2-distearoyl-sn-glycero-3-phosphocholine, DSPC), and 1,2-distearoyl-sn-glycero-3-phosphoethanolamine-N-[methoxy(polyethylene glycol)-2000] (ammonium salt) (PEG-DSPE) (DSPE-PEG). The nanoemulsion carries indomethacin (IND), a hydrophobic substance (Figure 1). The presence of the drug allows us to get to get more information about the solubilizing capacity of the nanoemulsions.

We have compared a high energy (HE) method (homogenization by ultrasounds) with a low energy (LE) method (spontaneous emulsification at high temperature). Sonication is capable of disrupting and intermingling the oil and aqueous phases into tiny oil droplets dispersed in water. Spontaneous emulsification involves pouring an organic phase (containing oil and surfactant) into an aqueous phase, which leads to the spontaneous formation of fine droplets due to rapid diffusion of the surfactant from the oil phase into the aqueous phase. The movement of the hydrophilic surfactant from the oil phase to the aqueous phase after mixing leads to the spontaneous formation of fine oil droplets at the oil-water boundary [11].

Our results demonstrate that both methods produce quite similar nanoemulsions. However, an important difference can be found concerning their long-term stability: the LE nanoemulsion is very stable when kept in quiescent conditions at room temperature or in the freezer, the usual conditions for storage.

## 2. Results

### 2.1. Characterization of the Nanoemulsions

Both kinds of nanoemulsions presented a monomodal distribution. Their *z*-average diameter was 156 ± 8 nm for the one obtained by the HE method, and 191 ± 1 nm for the nanoemulsion prepared according to the LE method. The corresponding polydispersity index was 0.07 ± 0.04 and 0.13 ± 0.02, respectively. Such values point out a remarkable monodispersity. Table 1 shows the comparative results on the content in iron, phospholipids, and IND.

**Figure 2:**
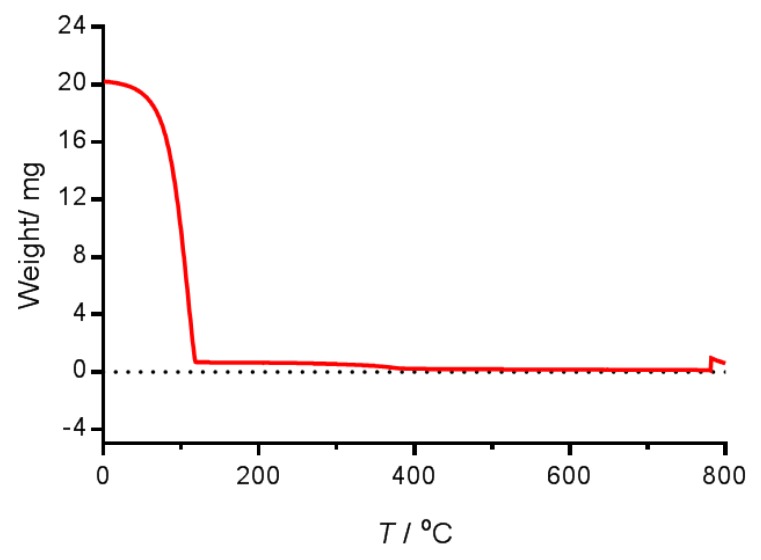
Thermogravimetric analysis of the nanoemulsion obtained by the LE method. The measurement was made from room temperature up to 1000 °C under nitrogen atmosphere (flux of 50 mL min^−1^) with a 10 °C min^−1^ heating rate.

Figure 2 shows the thermogravimetric analysis (TGA) of the nanoemulsion obtained by the LE method (the curve obtained with the nanoemulsion from the HE method was similar, as expected).

The TGA curve shows an important loss of weight (96.90%) in the temperature range from 30 °C to 145 °C due to the evaporation of the water content of the nanoemulsion. Another decrease in the mass profile (2.19%) occurred over a temperature range of 145–430 °C. This weight loss was due to the evaporation of surfactants and oils. Finally, the third weight loss (0.19%) between 800 °C and 1000 °C was a consequence of the transformation of γ-Fe_2_O_3_ to α-Fe_2_O_3_.

**Figure 3:**
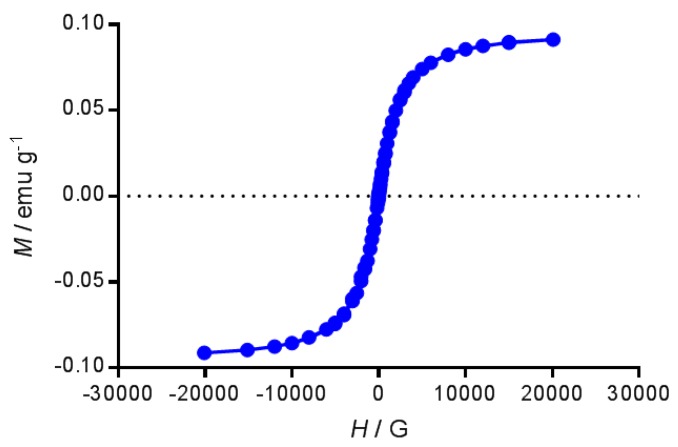
Mass magnetization as a function of the external magnet field for magnetic particles coated with oleic acid at 300 K. The external magnetic field was swept from 20,000 to −20,000 G and then back to 20,000 G.

The magnetization curve of the ferrofluid coated with oleic acid is shown in Figure 3. Measured magnetization was dependent on the magnitude of the applied film. Saturation magnetization (taken as magnetization at the field of 20,000 G) was 0.091 emu/g. This value corresponds to the suspension of magnetic particles in toluene. In relation to the solid particles, the saturation magnetization arises to 79.54 emu/g, which is lower than the theoretical value for the bulk Fe_3_O_4_ of 96.43 emu/g [27]. It is usual to obtain a softening of the magnetization due to the layer of stabilizer. It is interesting to remark that the magnetization curve lacks remnant magnetization at zero field, reflecting the superparamagnetic properties of the magnetic particles. The absence of hysteresis at room temperature is consistent with the nominal size of the magnetic nanoparticles, ~5 nm, which is associated with a superparamagnetic behavior [15].

### 2.2. In Vitro Release Assay

**Figure 4:**
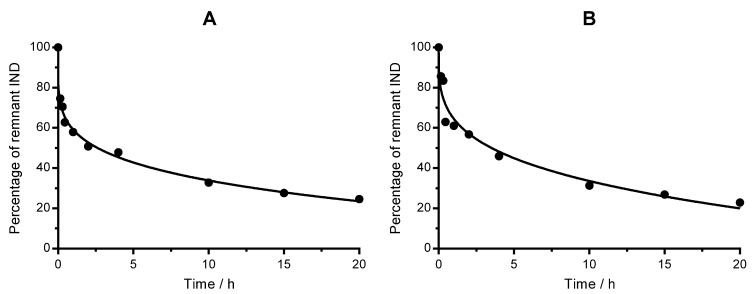
Time-dependent release kinetics of IND from nanoemulsions in Hepes buffer. Solid line is the fitting of the Korsmeyer-Peppas model to the experimental points. (**A**) High energy nanoemulsion, and (**B**) Low energy nanoemulsion.

Nanoemulsions were evaluated as potential controlled release drug delivery carriers. The release assay was carried out by dialysis. Release kinetics of IND from nanoemulsions was investigated in vitro for 20 h (Figure 4). As evidenced, a biphasic release pattern is observed for both nanoemulsions. There is a rapid initial release stage (up to 1 h), followed by a slower release phase (from 1 to 20 h). The quantity of drug released approaches 75.5% for HE nanoemulsion and 76.4% for LE nanoemulsion after 20 h.

### 2.3. Release Kinetics Modelling

In order to establish the model that explains the release of the drug from the nanoemulsion, five mathematical models were used to find which fitted to the release data: zero order, first order, Higuchi model, Hixson-Crowell model, and Korsmeyer-Peppas model [28]. The model with the higher determination coefficient (*R*^2^) was selected as the model that fitted the IND release data. As can be observed from Table 2, the Korsmeyer-Peppas model afforded the highest value of *R*^2^ for both nanoemulsions (*R*^2^ > 0.94, in both nanoemulsions).

This model is expressed by the equation $Q_{t}=Q_{0}-k_{\mathrm{KP}}t^{n}$, where *Q*_t_ is cumulative amount of drug released in time *t*, *Q*_0_ the initial amount of drug, t is the time, *k*_KP_ a constant called constant of the Korsmeyer-Peppas, and *n* is the diffusional release exponent. The kinetic model parameters fitting to the release data are displayed in Table 3.

In the Korsmeyer-Peppas model, the value of *n* characterized the mechanism of release. In this way, values of *n* ≤ 0.45 indicate that the mechanism of release is similar to a Fickian diffusion. Therefore, the influence of a possible interaction among drug molecules and the nanoemulsion components is minimal.

### 2.4. Long-Term Stability

**Figure 5:**
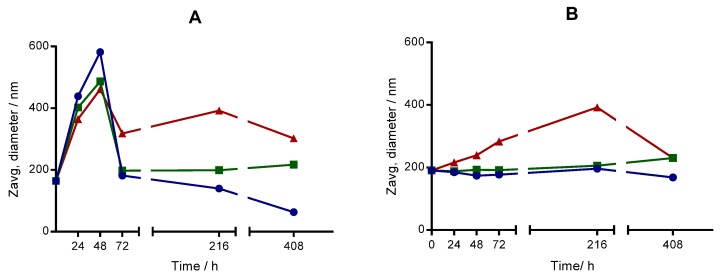
Long-term stability of nanoemulsions at 4 °C (blue dots), room temperature (green squares) and 37 °C (red triangles). Solid lines are guidelines. (**A**) High energy nanoemulsion, and (**B**) Low energy nanoemulsion.

For most commercial applications, it is important that nanoemulsion-based delivery systems remain physically stable throughout their shelf-life; i.e., there is little change in their particle size during storage. In this way, the stability of the magnetic nanoemulsions was examined at three temperatures (4, ~25 and 37 °C) by measuring the change of size in quiescent conditions. Figure 5 shows the profiles of the size changes as a function of time at three temperatures.

We clearly observe important differences between both kinds of nanoemulsions. The HE nanoemulsion underwent an important increase of the size even at 24 h after the preparation. At this time, the relative increase in the droplet size (Δ*d*) defined as Δ*d* = 100 × (*d_t_* − *d*_o_)/*d*_o_, where *d*_o_ and *d*_t_ are the mean droplet diameters at time *t* and 0, respectively, was 167% (4 °C), 145% (ambient temperature), and 122% (37 °C). The size achieved its maximal value after 48 h and then diminished in a fluctuating manner. The reduction of size can be attributed to the aggregation of nanoemulsion droplets and subsequent precipitation of the highest aggregates, since, although the density of the dispersed phase (oil) is lesser than the continuous phase (water), the density of the stabilizing layer around a nanoemulsion droplet is higher than the density of the oil. As other systems obtained by sonication, the first measurement of size gives higher values than the starting value. The reason is that the energy accumulated in the process overcomes the steric repulsion among the particles and the particles aggregate. Then, some or all of the aggregated particles settle down and they do not scatter the radiation any more. Contrarily, the LE nanoemulsion stored at 4 °C and ambient temperature kept approximately constant its size until 408 h. At 37 °C, the size increased steadily until 216 h. The important decrease observed at 408 h could be due to the coalescence and precipitation of droplets favored by higher temperature.

To obtain more information about the stability of the LE nanoemulsion, we studied the samples kept at 4 °C and ambient temperature for a minimum of two months. In both cases, the size was approximately 200 nm, and the polydispersity index never exceeded 0.13. This nanoemulsion was also kept at indicated temperatures in a diluted form (×10 in buffer solution) prior to storage. Neither size nor polydispersity index underwent significant changes in comparison with undiluted samples. This observation has important consequences for the practical application of this kind of nanoemulsion, since it can be stored in undiluted and in diluted forms.

### 2.5. Interaction with Proteins

**Figure 6:**
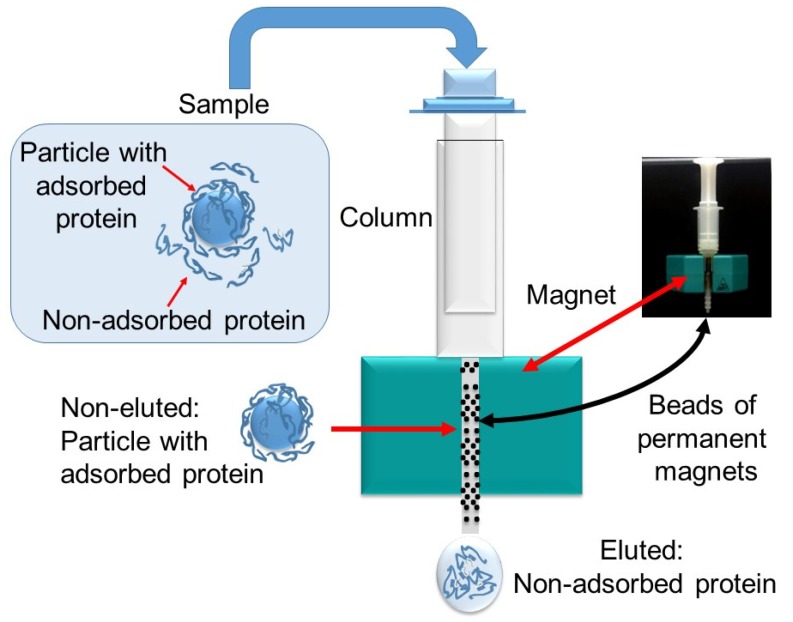
Experimental setup for determination of proteins bound to magnetic nanoemulsions. The MACS columns are composed of a spherical steel matrix; by inserting a column in a MACS separator, a high-gradient magnetic field is induced within the column, which retains the magnetic particles.

The surface of nanoparticles is covered by biomolecules upon coming into contact with biological systems. The interactions of the nanoparticles with the surrounding proteins can modify the properties of the nanoparticles when they circulate into the body. We performed a study to establish the extent of the interaction of our nanoemulsions first with a protein alone—bovine serum albumin (BSA)—and then with a more complex system—human plasma. When nanoemulsions were incubated with BSA, the size of the droplets did not undergo any significant change. After the desired time, the protein-associated magnetic droplets were run through a strong magnetic field using magnetic-activated cell sorting (MiniMACS), leading to the fixing of the magnetic droplets in the magnetic column (Figure 6). After washing with buffer, the flow-through fraction, which contains the protein bound to the droplets, was collected, and the protein content was determined. After transforming absorbance values in concentration (mg of protein by mL of nanoemulsion), the following values were obtained: 0.31 ± 0.04 mg/mL for HE nanoemulsion and 0.15 ± 0.03 mg/mL for LE nanoemulsion.

The incubation with human plasma afforded 0.007 ± 0.001 mg/mL for the nanoemulsion obtained by the HE method, while for the LE method the concentration was 0.041 ± 0.001 mg/mL. In this case, no significant changes in size were observed.

**Figure 7:**
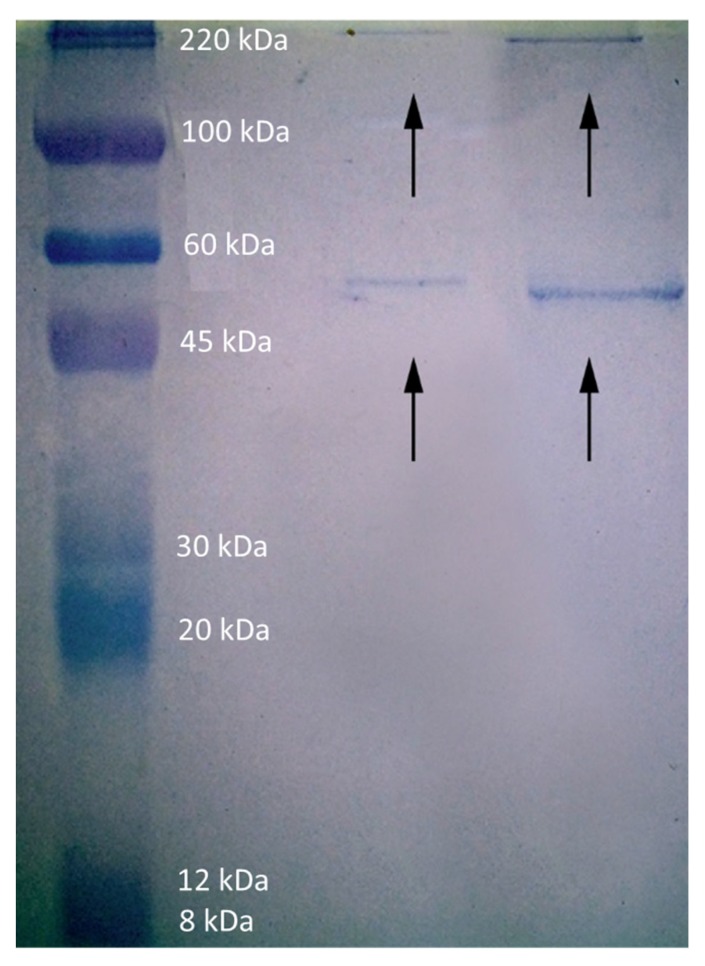
SDS-PAGE gel (12.5%) obtained from nanoemulsion-protein complexes after magnetic separation. The band on the left are the control proteins.

A polyacrylamide gel electrophoresis-sodium dodecyl sulfate (PAGE-SDS) electrophoresis was performed with the protein-associated magnetic droplets. After dying with Blue-Coomassie, two main bands appeared, approximately at the same level for both nanoemulsions. The upper band is located at the same level as the band of the marker corresponding to 220,000 Da, whereas the lower band is between the bands of the marker corresponding to 60,000 Da and 45,000 Da, but closer to 60,000 Da (Figure 7). From the figure, it is clear than the LE nanoemulsion droplets have adsorbed more protein than HE droplets do.

Blood plasma consists of over 3000 different proteins. Although all these proteins are capable of interacting with the nanoemulsion surface and form the so-called “protein corona” [29], the affinity between the surface and the proteins must be soft, and only the most abundant proteins in the plasma (albumin present at a concentration of ~44 mg mL^−1^, and immunoglobulin G at ~10 mg mL^−1^) remained adsorbed in a detectable amount after the repeated washings.

## 3. Discussion

Magnetic O/W nanoemulsions incorporating indomethacin were prepared using a low-energy or a high-energy method. Both kinds of nanoemulsions are extremely similar in relation to physicochemical properties, such as size, polydispersity, drug encapsulation, and iron content. The drug release from both nanoemulsions followed a pattern that can be quantified by the Korsmeyer-Peppas model. This fact indicates that the pharmacokinetics are in good accordance with Fickian diffusion. The main difference between both nanoemulsions lies in their storage stability. The nanoemulsion obtained at mild conditions, i.e., the LE nanoemulsion, was more stable than that obtained by sonication when stored in quiescent conditions at room temperature or in the freezer. Moreover, dilution did not affect the stability. Other important difference was the highest adsorption of proteins on the surface of the droplets of LE nanoemulsion. This different adsorption involves a different protein corona. The corona alters the surface composition and influences the nanomaterials’ biological identity as recognized by cells. In consequence, the behavior of the LE nanoemulsion will be quite different than the HE nanoemulsion droplets if it is administered intravenously. Since the adsorbed proteins control the interaction with cell membranes and the mechanism of cellular uptake, a potential future research direction should be the study of the interaction of the nanoemulsion with the biological milieu, including parameters as cytotoxicity, body distribution, and endocytosis into specific cells.

## 4. Materials and Methods

### 4.1. Materials

Extra virgin olive oil was from the Arbequina variety and had high oleic acid content (71.9% in weight). 1,2-Distearoyl-sn-glycero-3-phosphocholine (DSPC), iron oxide magnetic nanoparticles (5 mg/mL in toluene), and IND were purchased from Sigma-Aldrich (St. Louis, MO, USA); 1,2-distearoyl-sn-glycero-3-phosphoethanolamine-N-[methoxy(polyethylene glycol)-2000] (ammonium salt) (PEG-DSPE) was obtained from Avanti Polar Lipids (Alabaster, AL, USA). DSC and PEG-DSPE were dissolved in chloroform at 20 mg/mL. Olive oil was diluted in chloroform at 100 mg/mL. Spectra-Por^®^ Float-A-Lyzer^®^ G2 was purchased from Spectrum Labs (Rancho Dominguez, CA, USA). The aqueous phase used to prepare the nanoemulsions was Hepes sodium salt buffer solution (10 mM, pH 7.2). For sodium dodecyl sulfate-polyacrylamide gel electrophoresis (SDS-PAGE) the sample buffer NuPAGE^®^ LDS (Invitrogen, Carlsbad, CA, USA) was used. Double distilled water was used in the preparation of all the solutions. Organic solvents were of analytical grade.

### 4.2. Preparation of Nanoemulsions

#### 4.2.1. High Energy (HE) Method

The nanoemulsion was prepared by mixing 300 μL of extra virgin olive oil, 300 μL of DSPC, 1000 μL of PEG-DSPE and 75 µL of magnetic particles. The DSPC/PEG-DSPE molar ratio was 1.05. Then, the organic solvent was removed by evaporation at vacuum at 40 °C (Rotavapor R-3000, Büchi, Switzerland) for one hour. Once the solvent evaporated, 10 mL of Hepes buffer at 37 °C was added, followed by 15 min of agitation. The coarse emulsion obtained was sonicated in an UP200 St ultrasonic processor (Hielscher, Teltow, Germany) at a duty cycle of 70% and a frequency of 20 kHz, four times, for 5 min, with pauses of 1 min between sonications. Temperature reached in the sonication process was ≈50 °C. When the nanoemulsion contained IND, the drug dissolved in methanol (1 mg/mL) was incorporated (0.5 mL) to the mixture of surfactants and oil.

#### 4.2.2. Low Energy (LE) Method

The nanoemulsion was prepared with the same amounts used in the HE method via a spontaneous emulsification procedure [30]. In brief, the spontaneous emulsification was performed by titration of the organic phase (containing the indicated amounts of olive oil, DSPC, PEG-DSPE, and magnetic particles) at a rate of 2 mL/min into 10 mL of Hepes buffer warmed at 70 °C while continuously stirring the system with a magnetic stirrer. The magnetic stirring was maintained for 30 min to let the system reach equilibrium. Once the nanoemulsion was formed, it was centrifuged for 2 min at 1000 rpm. The supernatant was taken and sonicated in the ultrasonic processor two times for 10 s. When necessary, IND was incorporated in the nanoemulsion as described above.

#### 4.2.3. Particle Size Measurements

The particle size distribution and average particle diameter (*z*-average diameter) of nanoemulsions were measured by dynamic light scattering using a Zetasiser Nano ZS (Malvern, UK). The instrument determines the particle size from intensity-time fluctuations of a laser beam (633 nm) scattered from the sample at an angle of 90°. For the measurement, 50 µL of the nanoemulsion were diluted with Hepes buffer until 3 mL. Each individual measurement carried out at 25 °C was an average of 10 runs. Polydispersity index (PDI) is a dimensionless measure of the width of the size distribution calculated by the instrument. Samples were considered monodisperse when the PDI was lower than 0.2.

#### 4.2.4. Magnetic Measurements

The magnetic properties of the nanoemulsions were determined in a superconducting quantum interference device (SQUID) magnetometer (MPMS, Quantum Design, San Diego, CA, USA) at 300 K. For this, a few milligrams of the sample were lyophilized and the external magnetic field was swept from 20,000 to −20,000 G, and then back to 20,000 G. The saturation magnetization values were normalized to the mass of nanoparticles to yield the specsific magnetization, Ms (emu/g).

#### 4.2.5. Thermogravimetric Measurements

The thermogravimetric analysis of the nanoemulsions was carried out using a TGA/SDTA851e system (Mettler Toledo, Columbus, OH, USA) with a 10 °C/min heating rate under nitrogen atmosphere (50 mL/min). The measurement was made from room temperature up to 800 °C. 

#### 4.2.6. Lipid Determination

The lipid content based on the presence of phospholipids was determined by the Steward-Marshall method [31]. The calibration curve was made with different amounts of a chloroform solution of DSPC. The absorbance of the organic phase was read at 488 nm using the UV-24011PC UV-vis spectrophotometer (Shimadzu, Kyoto, Japan).

#### 4.2.7. Iron Determination

The iron content of the magnetic nanoemulsions was determined by the Kiwada method based on the determination of ferrous ion using *o*-phenanthroline [32]. The calibration curve was performed with a solution of Fe_3_O_4_ (Aldrich, Milwaukee, WI, USA) in hydrochloric acid (37%). The absorbance was measured at 509 nm. 

#### 4.2.8. IND Determination

Previous to the quantification of the IND encapsulated into the nanoemulsion, the method of Bligh and Dyer [33] was applied to extract the lipid from the nanoemulsion. Briefly, for each mL of nanoemulsion, the following solvents were added step by step, followed by gently vortexing after each addition: 3.75 mL of chloroform/methanol (1:2, *v*/*v*); 1.25 mL of chloroform, and 1.25 mL of distilled water. The mixture was then centrifuged at 1000 rpm at room temperature for 10 min, and two fractions were obtained. The bottom one containing the lipid phase was taken, and the volume was completed to 10 mL with chloroform:methanol (1:1, *v*/*v*). Finally, the absorbance was recorded at ~318 nm. A standard calibration curve was obtained for IND concentration calculation by fitting the measured absorbance with known drug concentration (100, 75, 50, 25, 10 µg/mL). The encapsulation efficiency (*EE*) was calculated using the following expression:

(1) $$EE\%=\frac{\mathrm{weight}\;\mathrm{of}\;\mathrm{IND}\;\mathrm{in}\;\mathrm{the}\;\mathrm{lipid}\;\mathrm{phase}}{\mathrm{weight}\;\mathrm{of}\;\mathrm{initial}\;\mathrm{IND}}\times 100$$

#### 4.2.9. In Vitro Release Assay

The release assay of IND was carried out using the Spectra-Por Float-A-Lyzer G2 dialysis device (Spectrum Labs, Rancho Dominguez, CA, USA) at room temperature. For the release experiment, 1 mL of the nanoemulsion was introduced into the dyalisis bag and placed in a vessel containing 100 mL of the receptor solution, Hepes buffer, under magnetic stirring. At predetermined time intervals (0, 15, 30, 45, 60, 120, 240, and 1200 min), 50 µL of sample were withdrawn. These samples were extracted according the Bligh and Dyer method, and the organic phase was read in a NanoDrop One/Onec spectrophotometer (Thermo Scientific, Rockford, IL, USA).

#### 4.2.10. Modelling of Release Kinetics

The release kinetics was determined by regression analysis of the in vitro release curves in five models: zero order, first order, Higuchi, Hixson-Crowell, and Korsmeyer-Peppas. The mathematical model that best expressed the kinetic release profile was selected based on the highest coefficient of determination (*r*^2^).

#### 4.2.11. Long-Term Stability Test

The long-term stability of nanoemulsions was assessed by measuring the change of droplet size and polydispersity by dynamic light scattering with time of storage. During the study, the nanoemulsions samples were aliquoted in vials, sealed, and kept at 37 °C, room temperature (~25 °C), and 4 °C. For the LE nanoemulsion, the study was carried out for two months at room temperature and 4 °C in the undiluted form and diluted 10 times with buffer solution.

#### 4.2.12. Interaction with Proteins

The study of the interaction of proteins was performed with bovine serum albumin (BSA) (Sigma-Aldrich, St. Louis, MO, USA) and with human plasma. Human plasma was obtained from the blood of healthy donors after obtaining informed consent. The blood was centrifuged to pellet red and white blood cells, and the plasma supernatant was pooled and stored at −80 °C. After thawing, the plasma was centrifuged at 20,000× *g* for 1 h at 4 °C to remove any residual protein precipitates. A protein concentration of 25 g/L was determined for the plasma. To determine the interaction, 0.5 mL of any nanoemulsion was mixed either with 1.5 mL of BSA (at 60 mg/mL in Hepes buffer) or with 1.5 mL of plasma. The mixing was incubated at 37 °C in quiescent conditions. Aliquots were withdrawn immediately after the mixing and at 1 h and 24 h of incubation. After 1 h, the size of the mixing was recorded. After 24 h, the size and the protein content was determined. The separation of free proteins from adsorbed proteins was carried out by magnetic separation (MiniMacs^™^, Miltenyi, Germany). The system consists of a magnetizable column matrix and a magnet. When located near the magnet, the column serves to create a high-gradient magnetic field. After washing the column with Hepes, 0.5 mL of magnetic nanoemulsion was passed through the column. Then, the retained material was washed with 0.5 mL of buffer to remove the non-retained material. Finally, to elute the retained material, the column was removed from the magnet, and the material was eluted with 0.5 mL of buffer with the aid of a plunger. With this eluate, the determination of the protein content was carried out by means of the Bradford method [34]. Briefly, 0.250 mL of eluate was mixed with 1.5 mL of Bradford reagent (Sigma, St. Louis, MO, USA); after mixing, the absorbance of the sample at 595 nm was read. BSA was used as standard (Protein Standard, Sigma, St. Louis, MO, USA). As a control, the same magnetic nanoemulsion incubated with buffer alone was used. The measurements were conducted in triplicate to ensure reproducibility of results.

#### 4.2.13. SDS-PAGE Electrophoresis

Proteins associated with the nanoemulsion droplets were mixed with protein solving buffer (15 µL of nanoemulsion with 5 µL of buffer) and boiled for 10 min at 90 °C. This solution was loaded onto a 12% polyacrylamide gel in a Mini-Protean Tetra device for 1-D vertical electrophoresis (Bio-Rad, Hercules, CA, USA) at a constant current of 20 mA per gel for 1 h. A molecular marker (ColorBurst^™^ Electrophoresis Marker, Sigma, St. Louis, MO, USA) was run in parallel. Proteins were fixed in 10% acetic acid for 1 h and subsequently visualized by staining with 0.1% Coomassie Brilliant Blue G-250 in 25% methanol for 24 h. 

## Figures and Tables

**Table 1:** Content in lipid, iron, and IND of the nanoemulsions (NE) obtained by high energy (HE) and low energy (LE) methods. The lipid is referred to the phospholipid content of the nanoemulsion. Values are the average ± standard deviation (*n* = 3).

Nanoemulsion	Lipid/mg mL^−1^	Iron/mg mL^−1^	IND/mg mL^−1^
HE	0.824 ± 0.054	0.250 ± 0.030	0.46 ± 0.07
LE	1.058 ± 0.040	0.210 ± 0.010	0.52 ± 0.08

**Table 2:** Mathematical models used with the release of drug data. $Q_{t}$: amount of drug released at time *t*; $Q_{0}$: amount of drug at time *t* = 0.

Nanoemulsion	Mathematical Model	*R*^2^	Equation
HE	Zero order First order Higuchi Hixson-Crowell Korsmeyer-Peppas	0.5827 0.8078 0.7996 0.7384 0.9918	Zero order $Q_{t}=Q_{0}-k_{o}t$ First order $Ln\;Q_{t}=ln\;Q_{0}-k_{1}t$ Higuchi $Q_{t}=Q_{0}-k_{H}t^{1/2}$ Hixson-Crowell $Q_{0}^{1/3}-Q_{t}{}^{1/3}=kt$ Korsmeyer-Peppas $Q_{t}=Q_{0}-k_{HP}t^{n}$
LE	Zero order First order Higuchi Hixson-Crowell Korsmeyer-Peppas	0.6219 0.8271 0.8340 0.7630 0.9458	

**Table 3:** Fitting parameters of the drug release kinetics to the Korsmeyer-Peppas model.

Nanoemulsion	Parameters	Values
HE	*k*_KP_/min^−*n*^	41 ± 3
	*n*	0.20 ± 0.02
LE	*k*_KP_/min^−*n*^	37 ± 7.1
	*n*	0.25 ± 0.05
